# Zoomed EPI-DWI of the Pancreas Using Two-Dimensional Spatially-Selective Radiofrequency Excitation Pulses

**DOI:** 10.1371/journal.pone.0089468

**Published:** 2014-03-03

**Authors:** Philipp Riffel, Henrik J. Michaely, John N. Morelli, Josef Pfeuffer, Ulrike I. Attenberger, Stefan O. Schoenberg, Stefan Haneder

**Affiliations:** 1 Institute of Clinical Radiology and Nuclear Medicine, University Medical Center Mannheim, Medical Faculty Mannheim – Heidelberg University, Mannheim, Germany; 2 Russell H. Morgan Department of Radiology and Radiological Science, Johns Hopkins University School of Medicine, Baltimore, Maryland, United States of America; 3 Siemens Healthcare Sector, Application Development, Erlangen, Germany; Oregon Health & Science University, United States of America

## Abstract

**Background:**

Implementation of DWI in the abdomen is challenging due to artifacts, particularly those arising from differences in tissue susceptibility. Two-dimensional, spatially-selective radiofrequency (RF) excitation pulses for single-shot echo-planar imaging (EPI) combined with a reduction in the FOV in the phase-encoding direction (i.e. zooming) leads to a decreased number of k-space acquisition lines, significantly shortening the EPI echo train and potentially susceptibility artifacts.

**Purpose:**

To assess the feasibility and image quality of a zoomed diffusion-weighted EPI (z-EPI) sequence in MR imaging of the pancreas. The approach is compared to conventional single-shot EPI (c-EPI).

**Material and Methods:**

23 patients who had undergone an MRI study of the abdomen were included in this retrospective study. Examinations were performed on a 3T whole-body MR system (Magnetom Skyra, Siemens) equipped with a two-channel fully dynamic parallel transmit array (TimTX TrueShape, Siemens). The acquired sequences consisted of a conventional EPI DWI of the abdomen and a zoomed EPI DWI of the pancreas. For z-EPI, the standard sinc excitation was replaced with a two-dimensional spatially-selective RF pulse using an echo-planar transmit trajectory. Images were evaluated with regard to image blur, respiratory motion artifacts, diagnostic confidence, delineation of the pancreas, and overall scan preference. Additionally ADC values of the pancreatic head, body, and tail were calculated and compared between sequences.

**Results:**

The pancreas was better delineated in every case (23/23) with z-EPI versus c-EPI. In every case (23/23), both readers preferred z-EPI overall to c-EPI. With z-EPI there was statistically significantly less image blur (p<0.0001) and respiratory motion artifact compared to c-EPI (p<0.0001). Diagnostic confidence was statistically significantly better with z-EPI (p<0.0001). No statistically significant differences in calculated ADC values were observed between the two sequences.

**Conclusion:**

Zoomed diffusion-weighted EPI leads to substantial image quality improvements with reduction of susceptibility artifacts in pancreatic DWI.

## Introduction

Diffusion-weighted imaging (DWI) plays an increasingly important role in the assessment of intra-abdominal pathology, both in oncologic and non-oncologic applications. DWI characterizes alterations in the random Brownian motion of water molecules. The technique has been successfully applied to the assessment of a variety of intraabdominal pathologies [1], [2], [3], [4] and utilized recently in the characterization of pancreatic lesions [5]. Specifically, lower apparent diffusion coefficient (ADC) values have been reported in pancreatic cancer and chronic pancreatitis [6], [7], [8], [9], whereas increased ADC values reflecting tissue edema and perfusion abnormalities have been shown in acute pancreatitis [10].

However, several limitations are encountered with the implementation of DWI within the abdomen. First, ADC values can vary as a result of acquisition techniques, b-values, the number of different b-values obtained, and field strength. Thus, the reproducibility of ADC measurements is a major concern [11], [12]. Moreover, quantitative ADC measurements in practice still remain in the realm of preclinical investigation and clinical trials. Second, DW imaging in the abdomen suffers from several artifacts. Single-shot spin-echo echo-planar imaging (EPI) is the most common sequence used for abdominal DWI. Echo-planar imaging is advantageous due to its short acquisition time but has many inherent problems such as chemical shift and susceptibility artifacts which result in ghosting and geometric distortion [13], [14]. The aforementioned artifacts are fundamentally caused by phase distortions which increase with longer gradient echo times and mimic the encoding of spatial information during image reconstruction. These effects degrade the achievable image quality, which is further limited by image blur due to the long EPI readout and corresponding low bandwidth per pixel in the phase encoding direction [15]. Such artifacts may be reduced through use of shorter echo trains [16] at the expense of longer scan times.

With the clinical availability of parallel radiofrequency (RF) transmission coils and the potential to utilize spatial information in an array during RF transmission, it is now possible to generate spatially tailored RF pulses. The combination of single-shot EPI with reduced-FOV imaging (i.e. “zoom”) in the phase-encoding direction and spatially-selective RF excitations results in a decreased number of acquisition steps and reduction in the EPI echo train [16], [17] without increase in scan time. In addition, with two independent transmit channels, the RF pulses can be optimized for a more homogeneous flip angle distribution through incorporation of B1 field information into the RF pulse calculation. The aim of this study is to evaluate the feasibility and clinical robustness of a zoomed diffusion-weighted EPI (z-EPI) sequence of the pancreas with comparison to a conventional single-shot EPI (c-EPI) sequence.

## Materials and Methods

### Ethics Statement

This retrospective study was approved by the ethical committee of our Institution (Medizinische Ethikkomission II der Medizinischen Fakultät Mannheim, Heidelberg Universität; Germany). The institutional review board waived the requirement of informed patient consent for this retrospective study. Information gathered on this population was performed in compliance with HIPAA guidelines.

### Study population

All patients who had undergone a MR-study of the pancreas on a parallel transmit MRI system from 12/04/2012 until 3/21/2013 were included. The patient population consisted of 23 patients (median age 56.4 years±15.1, range 23 – 78 years, 11 men, 12 women).

### MR imaging

All examinations were performed on a 3T whole-body MR system (MAGNETOM Skyra, Siemens Healthcare, Erlangen, Germany) equipped with a two-channel fully dynamic parallel transmit array (TimTX TrueShape, Siemens).

In all subjects, the protocol included a conventional DW-EPI acquisition using a parallel imaging factor of 2 and a zoomed EPI acquisition with a FOV reduced by a factor of 3; the latter using no further specific acceleration technique. The single-spoke sinc-excitation of the standard diffusion-weighted EPI sequence was replaced by a multi-spoke sinc-excitation using two-dimensional spatially-selective RF pulse with an echo-planar transmit trajectory similar to that described by Rieseberg et al [16]. Detailed imaging parameters are listed in Table 1.

**Table 1:** Imaging parameters.

	c-EPI	z-EPI
**TR/TE [ms]**	5900-8900/58-73	2900-4800/68-69
**Sequence type**	DW-SE-EPI	Zoomed DW-SE-EPI
**FOV [mm×mm]**	349-420/236-328	359-379/79-113
**Matrix**	192/90-112	200/31-54
**Slice thickness [mm]**	5	5
**Spatial resolution [mm^3^]**	2.6×2	2.5×1.9
**b-values**	50, 400, 800	50, 400, 800
**ZOOM factor Phase-enc**	none	3
**Parallel imaging**	GRAPPA 2	none
**Flip Angle**	90	90
**Fat suppression**	SPAIR	SPAIR
**Acquisition time [min]**	4.19	3.09

### Image Analysis

#### ADC Measurements

For each patient, the system-generated, mono-exponential ADC parameter maps were reviewed. For the ROI analysis, an OsiriX DICOM viewer (OsiriX 3.7.1; The OsiriX Foundation; Geneva, Switzerland) running on a commercially-available computer (MacPro, Apple, Cupertino, CA) was used. On each ADC parameter map, ROIs were placed manually over the anatomical distribution scanned. Average ADC values of the pancreas were calculated in the pancreatic head, the body, and the tail. Care was taken to measure only the intended region without including structural borders or prominent vascular structures within an anatomic segment. The mean ADC value of the ROI was recorded for further analysis. The average size of the ROI selected was 1.5 cm^2^. This procedure was repeated for both c-EPI and z-EPI patients in each of the 23 clinical patients.

#### Qualitative Image analysis

All patient data and acquisition parameters were removed from the data sets and presented in a blinded fashion and in random order to 2 board-certified radiologists with 6 and 8 years of experience in abdominal MRI. These radiologists evaluated all images independently using freely available software (OsiriX DICOM viewer 3.9.4, OsiriX Foundation; Geneva, Switzerland) on a MacPro workstation (Apple Inc, Cupertino, CA).

For each data set, each reader ranked c-EPI and z-EPI sequences in terms of delineation of the pancreas and overall scan preference. Additionally, each reader independently scored the following parameters of image quality using an ordinal Likert-type scale ranging from 1 to 4, with the lowest score (i.e. 1) indicating the best image quality: image blur, respiratory motion artifacts, and diagnostic confidence. Diagnostic confidence was defined in this case as the self-perceived ability of the reader to identify the pancreas as normal or abnormal and to characterize pancreatic pathology on a set of images. Additionally the readers ranked c-EPI and z-EPI sequences in terms of delineation of the pancreas and overall scan preference.

### Statistical Analysis

Statistical analyses were performed using standard statistical software (JMP 9.0, SAS Institute, Cary, North Carolina, USA). The Shapiro-Wilk W test was used to confirm a normality of the data distribution. Variables that are continuous are expressed as mean ± standard deviation. These were compared with either an independent t-test for normally distributed data or a Mann-Whitney U test for non-normally distributed data. Ordinal variables (image quality) are presented as medians with 25% to 75% interquartile ranges and were compared using the Kruskal-Wallis analysis of variance. P-values<0.05 were considered statistically significant.

## Results

All measurements were successfully performed. No data sets were excluded due to poor image quality.

### ADC Measurements

Mean ADC values of the different pancreas regions were calculated; the collected data is depicted in Table 2 and 3. In the head of the pancreas (p = 0.2), the pancreatic body (p = 0.4) and the pancreatic tail (p = 0.4), ADC values did not differ significantly between the two sequences.

**Table 2:** Individual ADC values (×10−3 s/mm^2^) for the 23 patients for both sequences.

	c-EPI			z-EPI		
Patient	Head	Body	Tail	Head	Body	Tail
**1**	1.22	1.39	1.19	1.22	1.29	1.18
**2**	1.22	1.25	1.02	1.25	1.35	1.08
**3**	1.03	1.08	1.08	1.00	1.04	1.09
**4**	0.92	1.09	1.07	0.96	1.17	1.03
**5**	1.25	1.23	1.22	1.19	1.23	1.28
**6**	0.90	1.23	0.99	0.98	1.28	0.98
**7**	1.03	1.27	1.04	1.14	1.20	1.01
**8**	1.07	1.02	1.09	1.11	1.03	1.13
**9**	1.20	0.95	0.84	1.15	1.04	0.98
**10**	1.36	1.30	0.97	1.32	1.30	1.07
**11**	0.95	1.23	1.10	0.92	1.13	1.10
**12**	1.29	1.57	1.22	1.27	1.54	1.17
**13**	1.05	1.18	1.24	1.03	1.18	1.17
**14**	1.50	1.23	1.23	1.52	1.25	1.31
**15**	1.29	1.25	1.17	1.24	1.27	1.29
**16**	1.45	1.43	1.46	1.54	1.46	1.40
**17**	1.48	1.41	1.39	1.54	1.49	1.36
**18**	1.03	1.01	0.89	1.07	1.12	0.98
**19**	1.41	1.07	1.40	1.36	1.07	1.31
**20**	1.49	1.63	1.74	1.35	1.45	1.30
**21**	0.96	1.23	1.03	1.10	1.24	1.12
**22**	0.86	0.88	0.99	0.88	0.85	0.99
**23**	1.24	1.01	1.02	1.25	1.30	1.11

**Table 3:** Mean ADC values (×10−3 s/mm^2^) and standard deviations in the pancreas for both sequences.

	c-EPI	z-EPI	
**Head**	1.2±0.2	1.2±0.2	p = 0.2
**Body**	1.2±0.3	1.2±0.2	p = 0.4
**Tail**	1.1±0.4	1.1±0.4	p = 0.4

### Qualitative Image analysis

**Figure 1:**
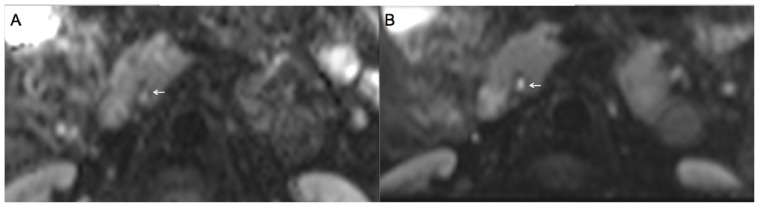
A 47-year old patient with c-EPI DWI (A) and z-EPI DWI (B) of the pancreas. z-EPI DWI demonstrates less distortion and a more homogeneous delineation of the pancreatic head and the main pancreatic duct (arrow).

**Figure 2:**
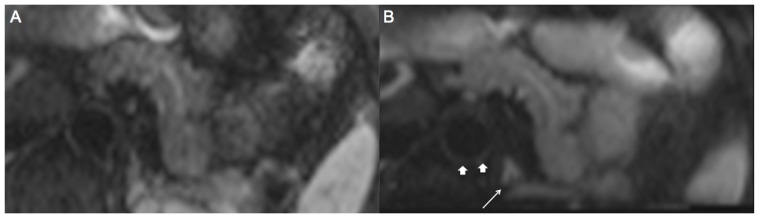
A 47-year old patient with c-EPI DWI (A) and z-EPI DWI (B) of the pancreas. z-EPI DWI demonstrates less distortion and a more homogeneous delineation of the pancreatic body. Also structures adjacent to the pancreas like the adrenal gland (thin arrow) and the aorta (arrows) are better delineated more sharply with z-EPI.

**Figure 3:**
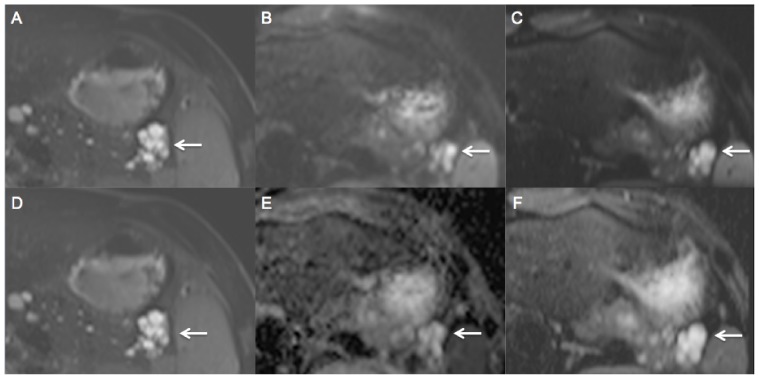
A 27-year old patient with serous cystadenoma in the pancreatic tail (arrow). T2 HASTE (A, D), c-EPI DWI (B, E) and z-EPI DWI (C, F) of the pancreas were acquired. z-EPI DWI (C) and zoomed ADC map (F) demonstrate a better delineation of the pancreas and the serous cystadenoma compared to c-EPI DWI (B) and conventional ADC map (E).

Readers 1 and 2 found the pancreas to be better delineated with z-EPI relative to c-EPI in every case (23/23) (Figure 1, 2 and 3). Inter-observer agreement was perfect in this regard (kappa = 1). Also structures adjacent to the pancreas, such as the adrenal glands and aorta, were depicted more sharply (Figure 2). For every patient scan (23/23), reader 1 and 2 preferred z-EPI overall to c-EPI. Inter-observer agreement was perfect in this regard (kappa = 1). With z-EPI there was statistically significantly less image blur (p<0.0001) and respiratory motion artifact compared to c-EPI (p<0.0001) (Table 4). Diagnostic confidence was statistically significantly better with z-EPI (p<0.0001).

**Table 4:** Image quality scores (median) of both sequences for different parameters.

	c-EPI	z-EPI	
**Image blur**	3	1	p<0.0001
**Respiratory motion artifact**	2	1	p<0.0001
**Diagnostic confidence**	3	1	p<0.0001

## Discussion

Abdominal DWI suffers from artifacts caused by distortions in the phase-encoding direction, which increase with larger field of views in the phase encoding direction and increased echo spacing. In a 2012 study, Rao et al demonstrated that 3T scanners incorporating the functionality of a 2-channel transmit array system enable improved image quality for DW images relative to 3T scanners without such functionality. Feeding the two ports of the transmit array with different amplitudes and phase relations (≠90 degrees) can potentially result in a more homogeneous B1 distribution and less signal shading due to B1 heterogeneity.

Recently, the utilization of RF pulses which differ not only in amplitude and phase, but also completely in the dynamic rf pulses for the individual transmit channels has been suggested [18], [19]. In this manner, it is possible to zoom-in in the phase-encoding direction, which leads to a decreased number of k-space acquisition lines and significantly shortens the length of the EPI echo train [16], [17]. The results of the present study indicate that the z-EPI technique offers considerable potential for overcoming some of the above-mentioned limitations of c-EPI techniques in abdominal imaging. Reduced FOV images lead to further improvements in image quality in terms of markedly reduced susceptibility artifacts. Significantly less image blur and respiratory motion artifact were observed with z-EPI compared to c-EPI, and the z-EPI images provided better delineation of the pancreas in all cases. Diagnostic confidence was likewise improved with z-EPI. As a result, the z-EPI was chosen as the preferred sequence in all cases.

The improved image quality with z-EPI could potentially help to identify malignant pancreatic neoplasms, for example intraductal papillary mucinous neoplasms (IPMN). In a 2013 study, Koung et al reported lower mean ADC values of malignant IPMN's relative to benign IPMN's and lower ADC values with invasive relative to non-invasive intraductal papillary mucinous carcinomas [20]. Qualitatively, the ability to predict invasive intraductal papillary mucinous carcinomas was improved; however, DWI failed to improve diagnostic accuracy for detection of malignant IMPN compared with the conventional image sets. The authors attributed this shortcoming to the inferior image quality of the DWI images secondary to susceptibility artifacts and spatial distortions. Further advances in abdominal DWI are therefore necessary, particularly with regard to susceptibility artifacts, in order to better characterize pancreatic tumors with the technique. This is particularly important for imaging at 3 T where susceptibility effects are doubled in magnitude relative to 1.5 T. These problems are also magnified within the pancreas, an area prone to susceptibility artifacts, as well as other anatomic regions such as the prostate and oral cavity. As the reproducibility of ADC values is a major concern, ADC values generated from z-EPI and c-EPI were compared in the pancreatic head, body, and tail in this work. No statistically significant difference in ADC values between z-EPI and c-EPI were found. For both sequences ADC values were similar to those published in previous studies [21], [22], [23].

The present study is not without limitations. Although performed in patients, in this feasibility study specific pancreatic pathologies were not specifically assessed with regard to determining the efficacy of DWI. This assessment represents a critical step prior to z-EPI becoming standard within the clinical routine. While the z-EPI approach does represent advantages relative to the imaging of particular structures, for example the z-EPI can be zoomed-in on the pancreas, detection and assessment of abnormalities outside the chosen field of view are limited. The incorporation of z-EPI within the clinical routine would thus exclude evaluation of structures outside the zoomed-in field of view. Thus, a combination of the two approaches may be warranted: 1) a c-EPI scan to screen the entire abdomen (possibly with lower spatial resolution) and 2) a z-EPI scan (with higher resolution) focused upon the anatomical structure or structures of interest. A stack of multiple z-EPI images from spatially shifted fields of view could also be combined. Further assessment of the z-EPI sequence is also needed in larger patient cohorts to confirm the findings presented in this work.

### Conclusion

Zoomed diffusion-weighted EPI of the pancreas leads to substantial image quality improvements and exhibits markedly reduced susceptibility and distortion artifacts relative to conventional EPI DWI.
